# Optimizing veteran-centered prostate cancer survivorship care: study protocol for a randomized controlled trial

**DOI:** 10.1186/s13063-017-1925-4

**Published:** 2017-04-18

**Authors:** Ted A. Skolarus, Tabitha Metreger, Soohyun Hwang, Hyungjin Myra Kim, Robert L. Grubb, Jeffrey R. Gingrich, Sarah T. Hawley

**Affiliations:** 1 VA HSR&D Center for Clinical Management Research, VA Ann Arbor Healthcare System, 2215 Fuller Road, Ann Arbor, MI 48105 USA; 2 Department of Urology, Dow Division of Health Services Research, University of Michigan, Ann Arbor, MI USA; 3 Department of Surgery (Urology), St. Louis VA Medical Center, Washington University School of Medicine, 915 North Grand Blvd., St. Louis, MO 63106 USA; 4 Department of Urology, VA Pittsburgh Healthcare System, University of Pittsburgh, 7180 Highland Drive, Pittsburgh, PA 15206 USA; 5 Department of Internal Medicine, University of Michigan, Ann Arbor, MI USA; 6 University of Michigan Center for Consulting for Statistics, Computing and Analytics Research, Ann Arbor, MI USA

**Keywords:** Prostatic neoplasms, Veterans, Self-care, Quality of life, Self-management, Survivorship, Health services

## Abstract

**Background:**

Although prostate cancer is the most common cancer among veterans receiving care in the Veterans Health Administration (VA), more needs to be done to understand and improve survivorship care for this large population. This study, funded by VA Health Services Research & Development (HSR&D), seeks to address the need to improve patient-centered survivorship care for veterans with prostate cancer.

**Methods/Design:**

This is a two-armed randomized controlled trial (RCT) with a target enrollment of up to 325 prostate cancer survivors per study arm (total anticipated n = 600). Patients will be recruited from four VA sites. Patient eligibility criteria include age range of 40–80 years, one to ten years post-treatment, and currently experiencing prostate cancer symptom burden. We will compare the “Building Your New Normal” program, a personally-tailored automated telephone symptom management intervention for improving symptom self-management to usual care enhanced with a non-tailored newsletter about symptom management. Primary outcomes include changes in symptom burden, bother, and health services utilization at five and 12 months after enrollment. Secondary outcomes include long-term psychosocial outcomes (e.g. subjective health, perceived cancer control). We will use multivariable regression analysis to evaluate the impact of the intervention on primary and secondary outcomes. We will conduct a process evaluation to understand the effective intervention components and explore possibilities for broader implementation and dissemination.

**Discussion:**

Our central hypothesis is that intervention group participants will have improved and more confident symptom self-management and prostate cancer quality of life following the intervention and that these outcomes will translate to more efficient use of health services. The study results will provide much needed information about how to optimize the quality of care, and life, of veteran prostate cancer survivors.

**Trial registration:**

ClinicalTrials.gov ID NCT01900561; Registered on 22 July 2013.

**Electronic supplementary material:**

The online version of this article (doi:10.1186/s13063-017-1925-4) contains supplementary material, which is available to authorized users.

## Background

Prostate cancer (PC) is the most commonly diagnosed cancer in US veterans [1]. Over 12,000 veterans will be diagnosed with PC in 2016 to join more than 150,000 veteran PC survivors in the Veterans Health Administration (VA). Because the majority of these veterans will live with the disease and its treatment side effects for many years, care during survivorship is an important priority for the VA. Nevertheless, there has been little systematic effort to understand the extent of veteran PC survivors’ symptom burden or how their symptom burden affects quality of life (QOL) and subsequent utilization of health services during survivorship. This gap in knowledge was highlighted in a recent review that recommended further efforts were needed to improve symptom burden in cancer survivors more broadly and understand relationships between symptom burden and service utilization [2].

There are three usual approaches to PC treatment including surgery (i.e. radical prostatectomy), radiation therapy (i.e. brachytherapy or external “beam” radiation), and observation (i.e. watchful waiting or active surveillance) [3, 4]. While some men choose observation initially, most eventually undergo some form of surgical, radiation, or hormonal treatment [5]. Each treatment can have distinct long-term side effects and many men experience ongoing problems with urination, sexual, hormonal (e.g. fatigue, depression), and bowel function (e.g. diarrhea and fecal incontinence) far beyond that experienced by age-matched controls [5–16].

**Fig. 1:**
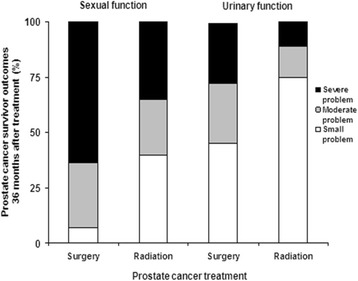
Symptom burden in PC survivors by treatment type

Many PC survivor studies from the general population demonstrate that such treatment-related sequelae significantly reduce disease-specific QOL (PC-QOL) for years following treatment [6, 8, 9, 17–22]. Studies also show the persistence of clinically significant PC treatment-related adverse effects well into survivorship and even up to ten years after diagnosis (Fig. 1) [14, 23–25]. Furthermore, there are important psychosocial consequences of treatment that can extend into survivorship, including worse perceptions of subjective health and cancer control (including worry about recurrence and confidence in one’s ability to deal with the cancer) [26–28]. Not surprisingly, PC symptoms worsen QOL and may lead to inefficient use of healthcare services (e.g. overuse of primary care for treatment complications such as urinary incontinence). The long-term persistence of PC symptoms is particularly unfortunate since many symptoms can be ameliorated or even eliminated through self-management or clinical intervention [29–63].

While the data documenting symptom burden in the general population of PC survivors is extensive, there has been no large-scale study of symptom burden in veteran survivors. Moreover, there have been no efforts to identify those survivors for whom better symptom self-management would translate into measurable improvements in QOL and more efficient use of healthcare services. Our pilot findings demonstrated that veteran PC survivors need and want assistance reducing their symptom burden and improving their disease-specific QOL. Yet, a system for patient-centered symptom assessment, self-management support, and connection to the appropriate healthcare providers for these survivors was lacking. Findings from our pilot studies also clearly support the feasibility of our proposed approach to combining automated symptom assessment with a tailored newsletter as a strategy for supporting symptom management among veteran PC survivors.

To address this gap in PC survivorship care in the VA, our team has embarked on a large randomized controlled trial (RCT) of a self-management support intervention. As we discuss below, the proposed project is based on pilot work, but will extend the intervention to survivors who are further from initial treatment. Using automated voice response (also called interactive voice response or IVR), we will assess ongoing symptoms (urinary, bowel, sexual, emotional, and general) in PC survivors and deliver self-management guidance to them in a series of tailored newsletters. We termed this intervention the “Building Your New Normal” (BNN) program. This low-cost tailored intervention will be compared with a non-tailored newsletter about self-management for a control group (BNN-control). We will evaluate the impact of the BNN intervention on primary outcomes of symptom burden and bother using the Expanded Prostate Cancer Index Composite (EPIC) measure, and secondary outcomes of self-management implementation, confidence in self-management, cancer control and outlook, self-efficacy in patient-physician interactions, and coping through conducting follow up surveys with participants at five and 12 months post enrollment. We will also assess use of PC-related health services through an audit of participants’ self-reported use and administrative data. Lastly, we will conduct a process evaluation to evaluate the BNN intervention’s full impact and prospects for broader dissemination and implementation.

### Conceptual framework

**Fig. 2:**
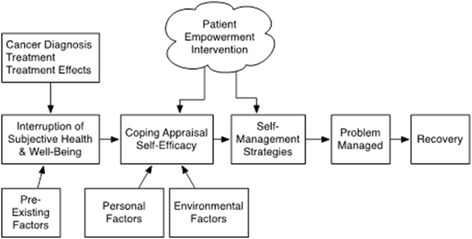
Conceptual model for self-management and recovery following cancer treatment

The conceptual framework for our intervention is the model of “Self-Management and Recovery following Cancer Treatment” proposed by Foster and Fenion (Fig. 2) [64], which describes the potential for self-management to have a positive impact on patient-reported outcomes for cancer survivors. The theoretical foundations for this framework are from social cognitive theory (e.g. the role of self-efficacy in health and wellbeing) and the transactional model of stress and coping (e.g. coping appraisal) [65, 66]. The framework is supported by a robust literature identifying the importance of self-care for chronic disease and prior studies demonstrating improved health outcomes and QOL among cancer patients as mediated through greater self-efficacy. In this framework, the diagnosis of cancer, cancer treatment, and treatment-related consequences are seen as decreasing a person’s sense of health and wellbeing. This disruption is followed by appraisal of the situation (i.e. coping appraisal) and appraisal of cancer-related self-efficacy to manage the situation. The person’s coping appraisal and self-efficacy then influence the type of self-management strategies that are used. The type of self-management strategies chosen influence whether the problem is managed effectively (i.e. a reduction in symptoms or perceived burden) and thus the degree of recovery of subjective health and potential improvement in cancer outlook [67]. The effects of cancer, coping appraisal, and self-efficacy are in turn influenced by pre-existing factors (e.g. age, gender, social roles, co-morbidities), personal factors (e.g. personality factors, mood states, general self-efficacy), and environmental factors (e.g. external support, healthcare services).

Within this framework, our proposed intervention will use a patient empowerment approach to specifically improve coping appraisal, self-efficacy for interacting with clinicians, and self-management strategies thereby potentially improving subjective health and cancer outlook. Empowerment is a patient-centered, collaborative approach that is tailored to match the patient’s health experience [68, 69]. Key elements of the empowerment-based approach include clarification of issues from the patient’s perspective (e.g. assessment of subjective “bother” as well as objective symptoms burden) and patient-directed goal setting and action planning (e.g. patient selection of priority symptom areas and self-management strategies). The intervention we propose to evaluate will also incorporate elements of cognitive behavioral therapy (CBT)-based self-management. CBT strategies are particularly relevant because they are specifically designed to simultaneously target reductions in symptoms as well as associated disability, emotional distress, and QOL [70]. During CBT, multiple skills are taught that address both cognitive processes (e.g. addressing overgeneralization, developing coping self-statements) and adoption of specific behavioral strategies (e.g. modified fluid intake, intentional partner communication). The success of CBT-based self-management interventions inside and outside the VA to reduce symptoms and improve function in patients with chronic conditions [71–73] lends support to our plan to apply these strategies to enhance self-management of post-treatment symptoms, coping, confidence, and cancer outlook among PC survivors.

Though there have been no comprehensive self-management interventions directed to help survivors reduce the negative consequences of PC treatment in everyday life, research suggests such interventions are likely to have a positive impact [74]. As shown in Table 1, each domain of PC treatment-related symptoms has corresponding self-management approaches with varying degrees of evidence. For example, urinary symptoms can be self-managed through a variety of approaches including emptying the bladder at regular intervals before it gets too full and pelvic floor (i.e. Kegel) exercises to help decrease urinary leakage. In fact, a RCT demonstrated a 50% decrease in incontinence episodes among PC survivors using pelvic floor muscle training and bladder control strategies [75]. A recent systematic review concluded that exercise improves incontinence, fatigue, body constitution, and QOL after treatment for PC [74]. Exercise among PC survivors is also associated with decreased mortality [76]. For sexual functioning after PC treatment, minimizing tobacco and excessive alcohol use, and communicating with partners about feelings and sex, are self-management strategies for improving sexual relationships and intimacy [77]. Avoiding spicy, greasy foods, coffee and alcohol, and staying well-hydrated may help limit the adverse bowel effects of radiation (i.e. radiation proctitis) among PC survivors [78]. However, there is no current systematic mechanism to share these strategies with veterans, despite their desire for knowledge of these approaches reported in our pilot survey.

**Table 1:** Symptom self-management strategies for PC survivors

	PC health domain			
	Urinary	Sexual	Bowel	Vitality, general health
Symptom	Leaked urine, increased frequency, dysuria	Erectile dysfunction, compromised masculinity, poor libido, poor communication, unable to engage in sexual activity	Diarrhea, fecal incontinence, increased urgency to defecate, increased frequency, pain, hematochezia	Depression, fatigue, hot flashes, weight gain, metabolic syndrome
Self-management strategy	Pelvic floor exercises (Kegels) [29–32] Bladder training (timed voiding) [33, 34] Fluid management (drinking) [35] Physical activity [36] Weight loss [37] Diet (avoid bladder irritants/caffeine) [38] Using a penile clamp [39] How to protect clothing and bedding [40]	Partner communication Making time for and enhancing intimacy [41] Creative intimacy (non-penile) [42–46] Having an orgasm without erection/avoiding climacturia [47–49] Reduce alcohol and tobacco [50, 51] Using a vacuum erection device [52–55] (NOTE: medical strategy following self-management approach)	Pelvic floor exercises (Kegels) [56, 57] Diet for softer stools (fiber) [58] Diet to avoid irritation Proper fluid intake Reducing painful bowel mvts Using OTC medications [58] Managing stress [59]	Decrease fatigue [60, 61] Exercise Improve mood/depression Deal with weight changes [62] Deal with hot flashes [63] Evaluate breast or nipple tenderness

### Specific aims

For these reasons, we aim to evaluate the ability of this intervention to improve key outcomes for veteran PC survivors. Based on our conceptual framework, the over-arching goal of this study is to determine whether an intervention using highly personalized automated telephone monitoring and self-management support calls paired with tailored print materials can effectively improve outcomes for veteran PC survivors. The study aims are as follows:

#### Aim 1: To conduct a randomized controlled trial among PC survivors with high symptom burden comparing the impact of a personalized intervention for improving symptoms and symptom self-management to non-personalized information

We hypothesize that relative to control participants, veterans in the BNN intervention group will: (1) have higher confidence about symptom self-management; and (2) report lower symptom burden and better disease-specific QOL at five and 12 months post enrollment. We further hypothesize that at the 12-month assessment point, the intervention group will have higher scores on two key psychosocial indicators (subjective health and perceived cancer outlook) than the control group.

#### Aim 2: To determine the intervention’s impact on the use of primary and specialty VA services

We hypothesize that relative to control participants, veterans in the BNN intervention group will have a higher rate of symptom-specific service use that is consistent with published expert and evidence-based recommendations for PC survivorship care 12 months after enrollment [79].

## Methods/Design

This is a two-armed RCT with an enrollment target of up to 325 PC survivors per arm (total anticipated n = 600). The intervention arm will receive the BNN intervention for improving symptom self-management. The control arm will receive enhanced usual care, consisting of one non-tailored newsletter describing self-management approaches in general. The Standard Protocol Items: Recommendations for Interventional Trials (SPIRIT) Checklist is presented as Additional file 1.

### Setting

This study is being conducted in four sites: the VA Ann Arbor Healthcare System (VAAAHS), the Louis Stokes Cleveland VA Medical Center (LSCVAMC), the VA Pittsburgh Healthcare System (VAPHS), and the St. Louis VA Medical Center (SLVAMC). Based on initial assessment of recruitment numbers, we added the LSCVAMC after the study began to meet recruitment goals. The sites offer significant numbers of potentially eligible survivors including racial/ethnic minority veterans (e.g. African Americans).

### Patient population and eligibility criteria

We will identify potential study participants using methods successfully used in prior studies of veteran PC survivors. Specifically, veterans who have been treated for PC by surgery or radiation therapy (using ICD-9 and HCPCS treatment codes) in the prior one to ten years will be identified from the VA National Patient Care Database and Corporate Data Warehouse data files. This eligibility was broadened from five years to ten years to help meet our recruitment goals. Patients treated with androgen deprivation therapy (ADT) will be eligible. To be eligible, patients must be aged 40–80 years and have a working cell or landline telephone. Patients will be ineligible if they are undergoing treatment for a non-PC, have dementia, or other significant mental impairment noted in their medical record.

### Potential risks and protections against them

The potential risks to patients participating in the study are minimal, as the intervention consists of participating in telephone assessments and receipt of tailored education or standard education similar to that available from other sources. Potential risks include loss of confidentiality and psychological distress. The psychological risks are related to possible coercion and potential emotions that may arise when responding to survey questions about health status and the challenges associated with managing symptoms of PC treatment including sexual health symptoms. Every effort will be made to minimize these risks. To avoid the possibility of coercion, an opt-out telephone number is provided and potential participants are told they are under no obligation to participate (i.e. it is a voluntary study) and that their care will not be affected by their decision to participate or not. Participants will be instructed to skip any survey question they do not wish to answer and are told that they may withdraw from the study at any time without penalty. To minimize the risk of loss of confidentiality, all data will be stored in secure locations accessible only to study team members.

It is possible the automated system and follow-up surveys may uncover certain symptoms that warrant medical attention. Two such possible symptoms include blood in stool and/or urine. If a study participant reports blood in stool and/or urine (or other concerning symptoms requiring the input of a physician), study staff will personally contact the participant via telephone to confirm the report. For all participants verifying current or recent symptoms, the appropriate provider will be notified. In the event a study participant has no assigned provider(s) at the VA appropriate for follow-up of reported concerning symptoms, study staff will encourage the participant to notify his primary care (or other) provider of the symptoms. Should the study participant express concerns or request additional information, or in the event the participant has no assigned community-based provider, the patient will be contacted by a study investigator who is a physician. In the event we are unable to reach participants by phone, study staff will send a letter by mail encouraging them to notify their provider of the reported symptoms.

Although we do not specifically ask about suicidal ideations, we do assess degree of depression in the EPIC measure and, as part of our tailoring for participants indicating they wanted help with depression symptoms, we included VA options to get help including talking with their primary care clinician and VA hotlines.

### Patient recruitment and consent

**Fig. 3:**
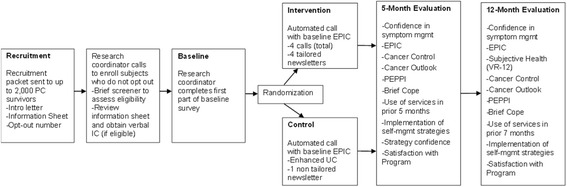
Study design

Patient recruitment will occur over a period of two years. (Note: recruitment started April 2015 and is projected to be completed by April 2017.) The project data manager reviews VA data files on a rolling basis to identify potentially eligible patients across all sites. Using the list compiled by the data manager, the project team sends a recruitment packet with an introductory letter and information sheet to potential participants (Fig. 3). The introductory letter includes a brief introduction to the study signed by the overall PI and the local site PI, plus a local or toll-free number to call for additional information or to request no further study-related contact (i.e. to “opt out”). The information sheet stands in for the informed consent document and includes a detailed description of the study and its potential benefits and risks. One week after the recruitment packet mailing, a research coordinator calls those veterans who have not declined further contact. Up to six attempts will be made. If patients have not been reached by the sixth attempt, no further contact is made.

Once a potentially eligible veteran has been reached, the research coordinator will briefly describe the study and ask if they would be interested in participating. Veterans who decline will not be contacted further; however, reason for refusal is requested and recorded when provided. Those who agree to participate will be asked to complete a brief screening tool to gauge desire for help managing symptoms in one of the four symptom areas: urinary, sexual, bowel, and general health. This screener asks veterans to indicate how much they would like help with managing symptoms in any of the four symptom areas (on a scale of 0 to 5, with 0 indicating patient does not want any help managing symptoms and 5 indicating patient would very much like help). Veterans scoring 1 or higher on any one symptom, as a measure of low function or high symptom burden, will be considered eligible to participate. Importantly, the screener is designed so that if the veteran wants help with a symptom—even if not really experiencing treatment-specific problems in that area—he is able to participate. The screener was designed to ensure that any PC survivor had the opportunity to participate in the study and work on a selected symptom domain of their choice through the tailored intervention. Each patient will receive an honorarium of $10 following randomization into one of the two arms that is sent to them in the mail. Any veteran who sounds depressed, or expresses depression or suicidal thoughts during the call with the research assistant is strongly encouraged to contact their PCP, and is given the telephone number for the suicide hotline. If a veteran expresses being unhappy with his care or with the VA specifically, the research coordinator offers to contact patient services and arranges a follow-up phone call to the veteran from the patient care representative.

### Patient baseline survey and randomization

The baseline survey for eligible patients is divided into two sections. The first half is conducted by the research coordinator immediately after screening questions and prior to randomization for all participants. This section verifies and collects patient demographics, confirms PC treatment type and diagnosis date, and assesses cancer outlook at the time of enrollment. We also administer the Perceived Efficacy in Patient-Physician Interactions (PEPPI) scale in this section. After completing this half of the baseline survey, participating patients are randomized by the computer into the BNN-intervention or BNN-control group. The randomization is stratified by original treatment type (surgery, radiation, or multiple types) to ensure there are equal proportions of patients by these factors in both arms. The randomization procedures are done via the computer console while the veteran is on the telephone. There is no way for randomization group to be determined prior to this point, nor is there any way to change the randomization group after it is completed.

After randomization, the second half of the baseline survey is initiated which consists of the standardized symptom burden questionnaire, the 26-item Expanded PC Index Composite (EPIC) instrument used to assess PC symptom burden and associated PC-QOL. To ensure the administration of the EPIC is standardized across participants, the EPIC will be administered to both intervention and control participants through the automated telephone system. The automated system will try to reach the veteran up to eight times over the course of four days following randomization to deliver EPIC. Even if the EPIC cannot be administered, the participant remains in the study per the intent-to-treat approach as discussed below. (See the “Data collection instruments” section below for more information on the PEPPI and EPIC.)

### Content and delivery of the BNN intervention

#### Intervention content

**Fig. 4:**
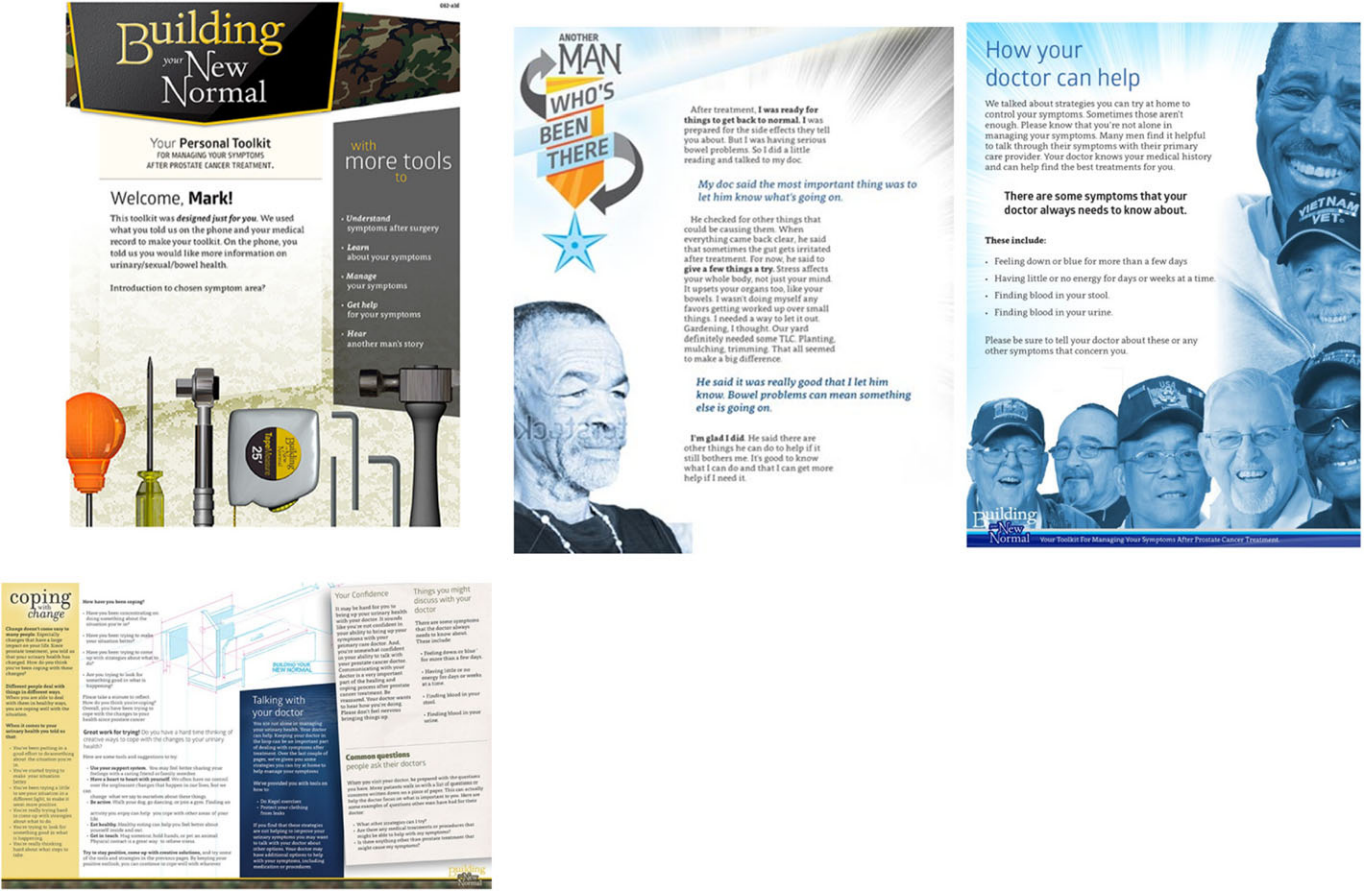
Personally tailored newsletter example

The BNN is a patient-centered intervention designed to improve confidence in symptom self-management, reduce symptom burden, and have subsequent positive impacts on subjective health (quality of life) and cancer outlook. The intervention is based on pilot work conducted by the study team and includes two components: (1) the automated voice response (IVR) telephone calls to assess symptoms and to offer participants the chance to choose a symptom to focus on (i.e. priority symptom); and (2) the tailored newsletter which is sent following the IVR that includes more detail about the symptom area chosen, as well as CBT-based approaches for coping with symptoms (see Fig. 4). Further detail regarding the delivery of the intervention components is provided below.

#### Intervention delivery

Veterans randomized to the BNN intervention will receive four automated phone calls over a three-month period: one call at study start and then once a month for three months. These calls will last approximately 15–25 min and include questions about symptoms, allow the veteran to identify a goal to work on, and help the veteran take steps towards reaching their goal and managing their symptoms. At the end of each call, the veteran will also have the option of listening to an audio testimonial tailored on a priority symptom area. Following each call, the veteran will receive a personalized newsletter that will provide more details regarding their symptoms and strategies to help them manage their symptoms. These recommendations are largely based on the Michigan Cancer Consortium Guidelines for the Primary Care Management of Prostate Cancer Post-Treatment Sequelae which include self-management, pharmacologic, medical, and surgical management strategies across the functional domains in EPIC [79]. Each newsletter will be four to eight pages in length. Example newsletter pages are illustrated in Fig. 4. The automated system will try to reach the veteran up to eight times over the course of four days (see Additional file 2: IVR call schedule). If the veteran has not completed the call by day 3 of the call window, study staff will make up to five or six attempts to contact the participant to verify the phone number and preferred call time. If the veteran has still not completed the call by day 5 of the call window, study staff will again make up to five or six attempts to contact the participant. Following each month’s reminder call, study staff will have the option to initiate additional automated calls should the veteran be agreeable. Veterans randomized to the BNN-control group will receive one four-page newsletter with educational information about symptoms and symptom management following completion of the baseline EPIC automated call.

### Follow-up assessments

Follow-up assessments will be completed by both groups at five and 12-months post enrollment. Beginning approximately four months following enrollment, initial attempts will be made by the study team to contact participants by phone to complete the five-month assessment and approximately 11 months following enrollment to complete the 12-month assessment. Up to five or six attempts will be made to contact participants for completion of follow-up assessments. Times to complete the surveys will be scheduled as desired by participants. The follow-up telephone interviews will take approximately 25–30 min based on piloting with the study team, with an anticipated range of 15–45 min depending on the participant’s ability to engage in the process. If participants are unable or unwilling to complete the survey by telephone, or cannot be reached by phone, a paper-and-pencil survey will be mailed to them with a return envelope. Participants who fail to return the paper survey within one month will be sent a second paper survey by mail. In addition, a reminder newsletter will be sent to participants one to two months prior to their expected 12-month follow-up. Follow-up surveys are also divided into two parts with the first part being administered by a research coordinator and the second half (the EPIC) being administered by the automated telephone system, again to ensure standardization of delivery of EPIC across groups.

## Data collection instruments

### Outcomes for Aim 1

**Fig. 5:**
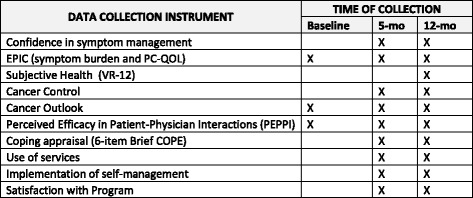
SPIRIT Figure: data collection instrument source and time of collection

The primary outcomes for Aim 1 (to evaluate the effectiveness of the BNN intervention for improving veteran-centered outcomes through an RCT) will be based on participant survey responses and our conceptual model (Fig. 2) for the anticipated impact of self-management on these response outcomes. The primary outcomes include: (1) confidence in symptom self-management as assessed at the five- and 12-month follow-ups; and (2) the symptom burden experienced by veteran PC survivors and associated PC-QOL, as assessed through the EPIC at baseline, five, and 12 months (see SPIRIT Figure: Fig. 5).

Secondary outcomes for Aim 1 include longer-term psychosocial outcomes, specifically subjective health (assessed at 12 months) and perceived cancer control and outlook (assessed at five and 12 months), both of which have been shown to be impacted by PC treatment [26–28], and, as noted earlier, both of which may be also be improved through effectively addressing coping and self-efficacy. We will also examine efficacy in patient–physician interactions and appraise coping as described below. Last, we will measure whether participants state they implemented a self-management approach and their overall satisfaction with the program.

#### Confidence in symptom self-management

To evaluate survivors’ confidence in addressing specific symptoms, we will use a five-question instrument developed for this study based on previous unpublished pilot work. Respondents indicate how confident they are that they: (1) understand why they experience symptoms after PC treatment; (2) know how to do things on their own (i.e. self-management) to address their symptoms; (3) know how their doctors can help with their symptoms; (4) can discuss their specific symptoms with their healthcare providers; and (5) feel knowledgeable about managing symptoms at home. Response options are based on a scale of 1–3 from “not at all” to “very confident.”

#### Symptom burden and QOL

We will use the EPIC instrument to assess symptom burden and PC-QOL at all three time points in this study. The EPIC has been widely used in PC survivorship research and practice including with automated telephone assessment by our group [6, 22, 80–82]. It is a 26-item measure that assesses symptom burden in four domains: urinary symptoms (nine items), bowel symptoms (six items), sexual symptoms (six items), and vitality (five items) (see Additional file 3: EPIC). Each domain has a subscale related to function and bother which together contribute to disease specific quality of life. These domains have good internal consistency (Chronbach’s alpha > 0.82 for each domain) and correlate highly with other established measures of QOL [80]. Each domain has a range of possible scores from 0 to 100, with lower scores indicating lower function in that domain (e.g. lower sexual health function) and lower QOL [80, 81, 83]. Thus, higher symptom burden, which reflects both function and bother, translates into lower EPIC scores. For purposes of this study we will consider a score of 70 or less to indicate “clinically meaningful symptom burden” for any one domain [84].

#### Subjective health

We will assess subjective health using three items from the VR-12 (SF-12 for veterans), an established measure of overall QOL that includes perceptions of one’s health that may be impacted by PC.

#### Cancer control and outlook

We will assess perceived cancer control and outlook using five items from a validated measure developed to examine the psychosocial impact of PC [26–28]. This measure, the Measuring Patients’ Perceptions of the Outcomes of Treatment for Early PC instrument by Clark et al., includes three domains related to confidence that one’s cancer is under control, worries about recurrence, and appraisals of one’s coping with PC. Cancer control will be assessed during the five- and 12-month follow-ups using three cancer control items from the instrument. Cancer outlook will be assessed at all three times points using the cancer outlook items from the instrument.

#### Efficacy in patient–physician interactions

Self-efficacy in patient–physician interactions will also be assessed at all three time points using a five-item short form version of the Perceived Efficacy in Patient-Physician Interactions (PEPPI) [85]. The PEPPI was developed to measure older patients’ self-efficacy in obtaining medical information and attention to their medical concerns from physicians.

#### Coping appraisal

We will assess participants’ coping during the five- and 12-month follow-up assessments using six items from the 28-item Brief Cope instrument. This instrument measures emotion-focused, problem-focused, and dysfunctional coping and has been used in cancer survivors [86].

### Outcomes for Aim 2

The primary outcome for Aim 2 will be use of VA health services related to PC survivorship care at the 12-month assessment point from enrollment. We will use the 12-month assessment point to ensure we capture any medication or service use related to PC symptom management that may occur after participants’ exposure to the intervention has ended. Because there are no VA guidelines for appropriate management of PC survivors, we will operationalize symptom-specific utilization consistent with published expert- and evidence-based guidelines for PC survivorship care [87]. These guidelines are meant to provide point-of-care recommendations for self-management, pharmacologic, medical, and surgical management strategies across the functional domains addressed in our study. We will use criteria mapping to operationalize utilization consistent with these guideline recommendations [88]. This process has been used extensively to develop sets of quality metrics using a set of recommendations, when evidence-based guidelines do not exist [89]. Specifically, for each symptom indicated by the participant at baseline, we will determine potentially appropriate use of services by mapping receipt of services at the 12-month follow-up to those outlined in the guideline recommendations. For analysis purposes, we will consider recommended survivorship care as “guideline-concordant” even though the guidelines are not fully evidence-based.

The outcomes for Aim 2 will be obtained from three VA data sources. We will collect pharmacy data from the VA Corporate Data Warehouse. National Patient Care Database records will be accessed for information including patient demographics, diagnoses, the date and type of utilization events, location of services (study site as well as clinic type), and the participants’ assigned primary care provider. To obtain information about device utilization, such as a penile clamp for incontinence control, we will use the National Patient Prosthetics Database from Patient Care Services. Having these records will ensure we can collect the most accurate device utilization. More broadly, we will use the datasets created through Aim 2 to assess primary and/or specialty care receipt 12 months post enrollment. To account for services potentially received outside of the VA, we will ask participants at both the five- and 12-month assessments to report the number of times over the prior period that they visited their VA or non-VA primary care physician and specialist (urologist, oncologist, radiation oncologist, or gastroenterologist). For each visit, we will ask them to indicate whether the visit was related to their PC symptom management.

### Analysis

#### Assessment of intervention uptake and intent-to-treat

A key consideration in this type of study is the degree to which those randomized to the intervention arm actively use the automated calls and tailored newsletters. Based on prior work, we anticipate drop-out to be small and that most participants randomized to the BNN intervention arm will complete at least three out of the four automated calls [90]. Prior studies using tailored newsletters also have shown that patient engagement is high, with one study conducted by our collaborator showing increased fruit and vegetable intake following delivery of tailored newsletters. Nevertheless, our primary analysis will be based on the intent-to-treat principle (ITT), [91] and will include all participants regardless of engagement in the intervention for both five-month and 12-month outcomes analyses. We will make every effort to obtain both five- and 12-month follow-up assessments of all randomized participants regardless of whether or not they completed the intervention sessions.

#### Completer analysis

In addition to ITT analysis, we will also do “completer” analysis where the completers will be defined as those who complete at least one automated call, and who complete the baseline and 5-month assessments. We will do similar completer analysis for long-term outcomes using 12-month assessments. If baseline characteristics of completers and non-completers are found to be different, we will also estimate the complier average causal effect [92]. This method uses the randomization process as an instrument to give an unbiased estimate (and standard error) of the causal effect of the intervention among those participants who actively participate. This approach is useful when trying to isolate the effect of the intervention itself independent of the effect of the intervention uptake rate on the overall intervention effect.

#### Engagement in intervention

Non-engagement in the intervention will include not only those with intermittent call completion, but also those who dropped out of the study or were lost-to-follow-up. We will compare dropout rates across intervention groups and we will use logistic regression models to explore predictors of dropout which include an indicator for intervention arm, key baseline characteristics (e.g. baseline symptom severity or measures of socioeconomic vulnerability), and interactions between group assignment and participant characteristics. The extent of missing data for baseline covariates as well as the missing outcome values at each follow-up assessment will be determined separately by study group. We will implement various methods including sensitivity analyses and multivariate sequential regression techniques to account for missing data and study dropout.

### Aim 1

#### Initial analyses and data verification

Baseline analysis (data verification) will include examining the distribution of all study variables to assess extreme values, missing data, variances, skewness, and type of distribution. Descriptive statistics will be used to describe the distribution of baseline variables in the two groups. We will use means, medians, and ranges for continuous variables such as age and baseline symptom scores, and we will use proportions for discrete variables. To assess balance between groups in the distribution of baseline characteristics, we will conduct baseline comparability of the two groups.

#### Aim 1 outcome analyses

Our primary outcomes for Aim 1 will be symptom scores using the EPIC measure and confidence in symptom self-management at five months. We will also evaluate these outcomes at the 12-month assessment point, but focus on the 5-month assessment for primary outcome assessment as it is closer to the completion of the intervention. We will also report by study group the percent of participants who reached EPIC symptom scores above 70 (i.e. “better symptom burden”) and better EPIC scores at both five and 12 months. We will evaluate the trends in symptom burden, and knowledge and information need over time by the two study groups. Results of these graphical analyses will be used to fine-tune the analytic course described below. Multiple regression analysis will be used to evaluate the primary outcomes in two stages. The first model will include an indicator for study group (intervention versus control) as the primary independent variable, adjusting for sites and treatment type received (radiation versus surgery). The second model will further adjust for baseline prognostic variables (i.e. age, education, income, race, and baseline symptom burden [EPIC score]).

The coefficient for the intervention group indicator will be used to evaluate the impact of the personalized automated PC symptom management program compared to non-tailored, standard information received by the control participants. We will repeat the analysis using each of the four symptom domains for EPIC. We will use the same analytic approach to evaluate confidence in symptom self-management and other secondary Aim 1 outcomes. We hypothesize that for all Aim 1 outcomes, intervention group survivors will have better scores at both five- and 12-month assessments relative to those in the control group (i.e. less symptom burden, higher confidence in symptom self-management, and better cancer outlook).

We will also assess the trajectory of symptoms over time. This will be done using a linear mixed-effects model with random effects for each subject, an indicator for intervention group, indicators of follow-up waves (five months and 12 months with baseline as reference) and interactions of follow-up times by intervention group to model potential changes in the intervention effect on symptoms soon (at five months) versus long (at 12 months) after the intervention is discontinued. If the contrast between five- and 12-month interaction is not significant, we will combine the post-enrollment times and estimate the time-averaged difference in outcomes between the two study groups. Analyses of other outcomes, such as self-management implementation and confidence, will also be assessed for the trajectory over time.

#### Aim 1 sample size and statistical power

We will use the minimal, clinically important difference in EPIC score for each of the four domains [84] to determine clinically meaningful differences in the between-group means for symptom scores by each domain. To detect a 0.33 standard deviation (SD) difference in symptom scores between participants receiving the automated PC symptom support versus standard information at 12 months with 90% power, we need 394 participants (197 per group), based on a regression analysis adjusting for baseline values with an α of 0.0125 with 0.5 as an assumed correlation between baseline and follow-up symptom scores. Note that the primary endpoint is five months, but we powered conservatively to detect a difference at 12 months and that the α value was chosen conservatively to detect symptom score difference in any of the four domains. The power will be higher to detect a larger than 0.33 SD between-group difference in symptom scores and also to detect the same difference with higher correlation than 0.5 between baseline and follow-up symptom values. Assuming conservatively 15% attrition at each assessment time, we propose to enroll 550 participants in total with an anticipated 397 participants to complete assessments at 12 months. The number of eligible PC patients at each site is sufficient for recruitment. For interval scale outcome measures, such as confidence in symptom self-management, the proposed sample size should give adequate power to detect at least 0.33 SD differences between groups.

### Aim 2

For Aim 2, we hypothesize that 12 months after enrollment, veterans in the automated PC symptom management intervention will have higher rates of use for PC survivorship services that are consistent with guideline recommendations than control participants. As described above, the primary outcome for Aim 2 will be use of symptom-specific guideline-concordant care assessed at 12 months. Using our global measure of use of any symptom-specific guideline-concordant care (yes/no), we will evaluate differences between intervention conditions using a logistic regression model including all participants and controlling for a priori baseline prognostic variables. We will then conduct additional logistic regression analysis within subgroups of patients with various symptom patterns at baseline. For these models, the primary outcome will be use of guideline-concordant care for a specific symptom (yes/no). We will control for veteran demographics (including age, race, education, and income), study site, type of treatment received (i.e. radiation, surgery, ADT). We will also explore the total amount of guideline-concordant services received in regression models appropriate for skewed count data (e.g. poisson or negative binomial models).

### Process evaluation

In addition to evaluating effectiveness of the intervention using standard RCT methods, rigorous and comprehensive process evaluation is essential to evaluate the full impact and prospects for broader dissemination and implementation [93]. We will conduct semi-structured qualitative interviews with up to 40 intervention participants (ten per site) and up to 40 stakeholders (four PCPs and four specialists at each site, plus interviews of VA stakeholders at the regional and national levels) to identify contextual factors that influence adoption and implementation [94] and to assess the patient-related factors impacting the intervention’s reach, engagement, satisfaction, and effectiveness [80, 95].

## Discussion

### Rationale for undergoing the trial

To our knowledge, the results of this RCT will be the first to compare easily scaled, tailored interventions for veteran PC survivors to standard interventions providing only basic non-tailored information on symptom management. Our pilot work provided compelling motivation for research to assist survivors with innovative services designed to increase access to self-management and medical management of their treatment-related symptoms. In addition, little is known about the association between PC-related symptom burden and service use in the VA, but our pilot study found that half of respondents did not know when to seek primary or specialty care to address their symptom(s). Further work is needed to understand how veterans’ symptom burden drives their use of health services and whether there is over- or under-use of care (or both). Moreover, VA leadership needs information about whether a symptom self-management intervention can improve appropriate service use for cancer survivors. Such information can provide better direction for survivors and their clinicians regarding how to optimize survivorship care both inside and outside of VA.

### Dissemination

Upon completion of the study and analysis of data, we plan to work with the primary care teams at each site in disseminating the study results. This will include presenting results to the local urology and primary care departments and nationally at research and operational meetings. Following completion of the 12-month assessment, participating patients will receive a newsletter containing some preliminary study results.

### Future directions

Should our analysis show the intervention is effective, we will work with the VA Office of Specialty Care Services to determine appropriate methods for broader dissemination. We will also plan to design and test an intervention for use in other healthcare delivery systems.

### Trial status

Patient recruitment started in April 2015 and will continue through the end of April 2017. As of February 23, 2017, the study has recruited 556 participants (target: 553–650).

## Additional files

Additional file 1: SPIRIT Checklist. (DOCX 23 kb)

Additional file 2: IVR call schedule. (DOCX 172 kb)

Additional file 3: EPIC. (DOC 120 kb)

## Abbreviations

ADT: Androgen deprivation therapy
BNN: Building Your New Normal
CBT: Cognitive behavioral therapy
CIRB: Central Institutional Review Board
EPIC: Expanded Prostate Cancer Index Composite
ITT: Intent-to-treat
IVR: Interactive voice response
LSCVAMC: Louis Stokes Cleveland VA Medical Center
PC: Prostate cancer
PEPPI: Perceived Efficacy in Patient-Physician Interactions
QOL: Quality of life
RCT: Randomized controlled trial
SLVAMC: St. Louis VA Medical Center
VA: Veterans Health Administration
VAAAHS: VA Ann Arbor Healthcare System
VAPHS: VA Pittsburgh Healthcare System

## References

[1] Zullig LL, Jackson GL, Dorn RA, Provenzale DT, McNeil R, Thomas CM (2012). Cancer incidence among patients of the U.S Veterans Affairs Health Care System. Mil Med.

[2] Robinson LL, Demark-Wahnefried W (2011). Cancer survivorship: focusing on future research opportunities. Cancer Epidemiol Biomarkers Prev.

[3] Wilt TJ, Shamliyan TA, Taylor BC, McDonald R, Kane RL (2008). Association between hospital and surgeon radical prostatectomy volume and patient outcomes: a systematic review. J Urol.

[4] Skolarus TA, Miller DC, Zhang Y, Hollingsworth JM, Hollenbeck BK (2010). The delivery of prostate cancer care in the United States: implications for delivery system reform. J Urol.

[5] Bill-Axelson A, Holmberg L, Ruutu M, Garmo H, Stark JR, Busch C (2005). Radical prostatectomy versus watchful waiting in early prostate cancer. N Engl J Med.

[6] Watson E, Shinkins B, Frith E, Neal D, Hamdy F, Walter F, Weller D, Wilkinson C, Faithfull S, Wolstenholme J, Sooriakumaran P, Kastner C, Campbell C, Neal R, Butcher H, Matthews M, Perera R, Rose P. Symptoms, unmet needs, psychological well-being and health status in survivors of prostate cancer: implications for redesigning follow-up. BJU Int. 2016;117(6B):E10–9. doi:10.1111/bju.13122. Epub 2015 May 23. 10.1111/bju.13122 25818406

[7] Alemozaffar M, Regan MM, Cooperberg MR, Wei JT, Michalski JM, Sandler HM (2011). Prediction of erectile function following treatment for prostate cancer. JAMA.

[8] Dandapani SV, Sanda MG (2008). Measuring health-related quality of life consequences from primary treatment for early-state prostate cancer. Semin Radiat Oncol.

[9] Johansson E, Steineck G, Holmberg L, Johansson JE, Nyberg T, Ruutu M (2011). Long-term quality of life outcomes after radical prostatectomy or watchful waiting: The Scandinavian Prostate Cancer Group-4 randomized trial. Lancet Oncol.

[10] Johansson E, Bill-Axelson A, Holmberg L, Onelov E, Johansson JE, Steineck G (2009). Time, symptom burden, androgen deprivation, and self-assessed quality of life after radical prostatectomy or watchful waiting: the Randomized Scandinavian Prostate Cancer Group Study Number 4 (SPCG-4) clinical trial. Eur Urol.

[11] Steineck G, Helgesen F, Adolfsson J, Dickman PW, Johansson JE, Norlen BJ (2002). Quality of life after radical prostatectomy or watchful waiting. N Engl J Med.

[12] Penson DF, Litwin MS (2003). Quality of life after treatment for prostate cancer. Curr Urol Rep.

[13] Kerleau C, Guizard AV, Daubisse-Marliac L, Heutte N, Mercier M, Grosclaude P, Joly F; French Network of Cancer Registries (FRANCIM). Long-term quality of life among localised prostate cancer survivors: QALIPRO population-based study. Eur J Cancer. 2016;63:143–53. doi:10.1016/j.ejca.2016.05.020. Epub 2016 Jun 15. 10.1016/j.ejca.2016.05.020 27318002

[14] Gore JL, Kwan L, Lee SP, Reiter RE, Litwin MS (2009). Survivorship beyond convalescence: 48-month quality-of-life outcomes after treatment for localized prostate cancer. J Natl Cancer Inst.

[15] Hoffman RM, MacDonald R, Wilt TJ (2004). Laser prostatectomy for benign prostatic obstruction. Cochrane Database Syst Rev.

[16] Reeve BB, Stover AM, Jensen RE, Chen RC, Taylor KL, Clauser SB (2012). Impact of diagnosis and treatment of clinically localized prostate cancer on health-related quality of life for older Americans: a population-based study. Cancer.

[17] Ferrer M, Suarez JF, Guedea F, Fernandez P, Macias V, Marino A (2008). Health-related quality of life two years after treatment with radical prostatectomy, prostate brachytherapy, or external beam radiotherapy in patients with clinically localized prostate cancer. Int J Radiat Oncol Biol Phys.

[18] Potosky AL, Legler J, Albertsen PC, Stanford JL, Gilliland FD, Hamilton AS (2000). Health outcomes after prostatectomy or radiotherapy for prostate cancer: results from the Prostate Cancer Outcomes Study. J Natl Cancer Inst.

[19] Potosky AL, Davis WW, Hoffman RM, Stanford JL, Stephenson RA, Penson DF (2004). Five-year outcomes after prostatectomy or radiotherapy for prostate cancer: the prostate cancer outcomes study. J Natl Cancer Inst.

[20] Wei JT, Dunn RL, Sandler HM, McLaughlin PW, Montie JE, Litwin MS (2002). Comprehensive comparison of health-related quality of life after contemporary therapies for localized prostate cancer. J Clin Oncol.

[21] Harrington CB, Hansen JA, Moskowitz M, Todd BL, Feuerstein M (2010). It’s not over when it’s over: long-term symptoms in cancer survivors -- a systematic review. J Psychiatry Med.

[22] Wei JT, Dunn RL, Marcovich R, Montie JE, Sanda MG (2000). Prospective assessment of patient reported urinary continence after radical prostatectomy. J Urol.

[23] Miller DC, Wei JT, Dunn RL, Montie JE, Pimentel H, Sandler HM (2006). Use of medications or devices for erectile dysfunction among long-term prostate cancer treatment survivors: potential influence of sexual motivation and/or indifference. Urology.

[24] Taylor KL, Luta G, Miller AB, Church TR, Kelly SP, Muenz LR (2012). Long-term disease-specific functioning among prostate cancer survivors and non-cancer controls in the prostate, lung, colorectal, and ovarian cancer screening trial. J Clin Oncol.

[25] Pardo Y, Guedea F, Aguilo F, Fernandez P, Macias V, Marino A (2010). Quality-of-life impact of primary treatments for localized prostate cancer in patients without hormonal treatment. J Clin Oncol.

[26] Clark JA, Rieker P, Propert KJ, Talcott JA (1999). Changes in quality of life following treatment for early prostate cancer. Urology.

[27] Clark JA, Talcott JA (2006). Confidence and uncertainty long after initial treatment for early prostate cancer: survivors’ views of cancer control and the treatment decisions they made. J Clin Oncol.

[28] Clark JA, Inui TS, Silliman RA, Bokhour BG, Krasnow SH, Robinson RA (2003). Patients’ perceptions of quality of life after treatment for early prostate cancer. J Clin Oncol.

[29] Messer KL, Hines SH, Raghunathan TE, Seng JS, Diokno AC (2007). Self-efficacy as a predictor to PFMT adherence in a prevention of urinary incontinence clinical trial. Health Educ Behav.

[30] Zhang AY, Fu AZ, Moore S, Zhu H, Strauss G, Kresevic D, Klein E, Ponsky L, Bodner DR. Is a behavioral treatment for urinary incontinence beneficial to prostate cancer survivors as a follow-up care? J Cancer Surviv. 2017;11(1):24–31. doi:10.1007/s11764-016-0557-0. Epub 2016 Jun 24. 10.1007/s11764-016-0557-0 PMC8101047 27341843

[31] Ribeiro LH, Prota C, Gomes CM, de Bessa J, Boldarine MP, Dall’Oglio MF (2010). Long-term effect of early postoperative pelvic floor biofeedback on continence in men undergoing radical prostatectomy: a prospective, randomized, controlled trial. J Urol.

[32] Hunter KF, Glazener CM, Moore KN (2007). Conservative management for postprostatectomy urinary incontinence. Cochrane Database Syst Rev.

[33] Dougherty MC, Dwyer JW, Pendergast JF, Boyington AR, Tomlinson BU, Coward RT (2002). A randomized trial of behavioral management for continence with older rural women. Res Nurs Health.

[34] Wallace SA, Roe B, Williams K, Palmer M. Bladder training for urinary incontinence in adults. Cochrane Database Syst Rev (Online). 2004;(1):CD001308. 10.1002/14651858.CD001308.pub2 PMC7027684 14973967

[35] Milne JL (2004). Behavioral therapies at the primary care level: the current state of knowledge. J Wound Ostomy Cont Nurs.

[36] Wolin K, Luly J, Sutcliffe S, Andriole G, Kibel A (2010). Risk of urinary incontinence following prostatectomy: the role of physical activity and obesity. J Urol.

[37] Subak LL, Johnson C, Whitcomb E, Boban D, Saxton J, Brown JS (2002). Does weight loss improve incontinence in moderately obese women?. Int Urogynecol J Pelvic Floor Dysfunct.

[38] Arya LA, Myers DL, Jackson ND (2000). Dietary caffeine intake and the risk for detrusor instability: a case-control study. Obstet Gynecol.

[39] Campbell SE, Glazener CM, Hunter KF, Cody JD, Moore KN (2012). Conservative management for postprostatectomy urinary incontinence. Cochrane Database Syst Rev (Online).

[40] Fader M, Cottenden A, Getliffe K, Gage H, Clarke-O’Neill S, Jamieson K (2008). Absorbent products for urinary/faecal incontinence: a comparative evaluation of key product designs. Health Technol Assess.

[41] Manne SL, Kissane DW, Nelson CJ, Mulhall JP, Winkel G, Zaider T (2011). Intimacy-enhancing psychological intervention for men diagnosed with prostate cancer and their partners: a pilot study. J Sex Med.

[42] Manne S, Mulhall JP, Incrocci L, Goldstein I, Rosen R (2011). Restoring intimacy in relationships affected by cancer. Cancer and Sexual Health.

[43] Manne S, Kissane D, Zaider T, Nelson C, Winkel G (2010). Intimacy-enhancing intervention for couples coping with localized prostate cancer. Ann Behav Med.

[44] Brotto LA, Yule M, Breckon E (2010). Psychological interventions for the sexual sequelae of cancer: a review of the literature. J Cancer Surviv.

[45] Manne S, Badr H (2008). Intimacy and relationship processes in couples’ psychosocial adaptation to cancer. Cancer.

[46] Manne S, Ostroff J, Rini C (2004). The interpersonal process model of intimacy: The role of self-disclosure, partner disclosure, and partner responsiveness in interactions between breast cancer patients and their partners. J Fam Psychol.

[47] Mehta A, Deveci S, Mulhall JP (2013). Efficacy of a penile variable tension loop for improving climacturia after radical prostatectomy. BJU Int.

[48] Fenner A (2011). Prostate cancer: postprostatectomy climacturia. Nat Rev Urol.

[49] Sighinolfi MC, Rivalta M, Mofferdin A, Micali S, De Stefani S, Bianchi G (2009). Potential effectiveness of pelvic floor rehabilitation treatment for postradical prostatectomy incontinence, climacturia, and erectile dysfunction: a case series. J Sex Med.

[50] Harte CB, Meston CM (2012). Association between smoking cessation and sexual health in men. BJU Int.

[51] Meldrum DR, Gambone JC, Morris MA, Esposito K, Giugliano D, Ignarro LJ (2012). Lifestyle and metabolic approaches to maximizing erectile and vascular health. Int J Impot Res.

[52] Engel JD (2011). Effect on sexual functoin of a vacuum erection device post-prostatectomy. Can J Urol.

[53] Raina R, Pahlajani G, Agarwal A, Jones S, Zippe C (2010). Long-term potency after early use of a vacuum erection device following radical prostatectomy. BJU Int.

[54] Dalkin BL, Christopher BA (2007). Preservation of penile length after radical prostatectomy: early intervention with a vacuum erection device. Int J Impot Res.

[55] Hellstrom WJ, Montague DK, Moncada I, Carson C, Minhas S, Faria G (2010). Implants, mechanical devices, and vascular surgery for erectile dysfunction. J Sex Med.

[56] Norton C, Cody JD (2012). Biofeedback and/or sphincter exercises for the treatment of faecal incontinence in adults. Cochrane Database Syst Rev.

[57] Mishra SI, Scherer RW, Snyder C, Geigle PM, Berlanstein DR, Topaloglu O (2012). Exercise interventions on health-related quality of life for people with cancer during active treatment. Cochrane Database Syst Rev.

[58] Gibson RJ, Keefe DM, Lalla RV, Bateman E, Blijlevens N, Fijlstra M (2012). Systematic review of agents for the management of gastrointestinal mucositis in cancer patients. Support Care Cancer.

[59] Konturek PC, Brzozowski T, Konturek SJ (2011). Stress and the gut: pathophysiology, clinical consequences, diagnostic approach and treatment options. J Physiol Pharmacol.

[60] Savard J, Hervouet S, Ivers H (2013). Prostate cancer treatments and their side effects are associated with increased insomnia. Psychooncology.

[61] Segal RJ, Reid RD, Courneya KS, Malone SC, Parliament MB, Scott CG (2003). Resistance exercise in men receiving androgen deprivation therapy for prostate cancer. J Clin Oncol.

[62] Davies NJ, Batehup L, Thomas R (2011). The role of diet and physical activity in breast, colorectal, and prostate cancer survivorship: a review of the literature. Br J Cancer.

[63] Morrow PKH, Mattair DN, Hortobagyi GN (2011). Hot flashes: a review of pathphysiology and treatment modalities. Oncologist.

[64] Foster C, Fenion D (2011). Recovery and self-management support following primary cancer treatment. Br J Cancer.

[65] Bandura A (1982). Self-efficacy mechanism in human agency. Am Psychol.

[66] Bandura A (1977). Social Learning Theory.

[67] Torbit LA, Albiani JJ, Crangle CJ, Latini DM, Hart TL (2015). Fear of recurrence: The importance of self-efficacy and satisfaction with care in gay men with prostate cancer. Psychooncology.

[68] Anderson M, Funnell MM (2004). Empowerment and self-management of diabetes. Clin Diab.

[69] Arnold MS, Butler PM, Anderson RM, Funnell MM, Feste C (1995). Guidelines for facilitating a patient empowerment program. Diabetes Educ.

[70] Turk DC, Meichenbaum D, Genest M (1983). Pain and behavioral medicine: a cognitive behavioral approach.

[71] Piette JD, Richardson C, Himle J, Duffy S, Torres T, Vogel M (2011). A randomized trial of telephonic counseling plus walking for depressed diabetes patients. Med Care.

[72] Piette JD (2005). Use of CBT in a walking program for veterans with diabetes and depression. Psychiatr Serv.

[73] Price JR, Couper J (2000). Cognitive behavior therapy for adults with chronic fatigue syndrome. Cochrane Database Syst Rev.

[74] Baumann FT, Zoph EM, Bloch W (2011). Clinical exercise interventions in prostate cancer patients -- a systematic review of randomized controlled trials. Support Care Cancer.

[75] Goode PS, Burgio KL, Johnson TM, Clay OJ, Roth DL, Markland AD (2011). Behavioral therapy with or without biofeedback and pelvic floor electrical stimulation for persistent postprostatectomy incontinence. JAMA.

[76] Kenfield SA, Stampfer MJ, Giovannucci E, Chan JM (2011). Physical activity and survival after prostate cancer diagnosis in the health professionals follow-up study. J Clin Oncol.

[77] Meldrum DR, Gambone JC, Morris MA, Ignarro LJ (2010). A multifaceted approach to maximize erectile function and vascular health. Fertil Steril.

[78] National Cancer Institute. http://www.cancer.gov/cancertopics/pdq/supportivecare/gastrointestinalcomplications/HealthProfessional/. Accessed 11 Apr 2017.

[79] Michigan Cancer Consortium Prostate Cancer Action Committee. Guidelines for Primary Care Management of Prostate Cancer Post-Treatment Sequelae. 2009. http://www.michigancancer.org/PDFs/AboutTheMCC/MCCMeetings/BoDMtg/MCCMtgMinutes/2013/09182013/PCAC.Skolarus.09182013.pdf. Accessed 13 Apr 2017.

[80] Chang P, Szymanski KM, Dunn RL, Chipman JJ, Litwin MS (2011). Expanded prostate cancer index composite for clinical practice: development and validation of a practical health related quality of life instrument for use in the routine clinical care of patients with prostate cancer. J Urol.

[81] Wei JT, Dunn RL, Litwin MS, Sandler HM, Sanda MG (2000). Development and validation of the expanded prostate cancer index composite (EPIC) for comprehensive assessment of health-related quality of life in men with prostate cancer. Urology.

[82] Wei JT, Montie JE (2000). Comparison of patients’ and physicians’ rating of urinary incontinence following radical prostatectomy. Semin Urol Oncol.

[83] Sanda MG, Dunn RL, Michalski J, Sandler HM, Northouse L (2008). Quality of life and satisfaction with outcome among prostate cancer survivors. N Engl J Med.

[84] Dunn RL, Sanda MG, Wei JT (2009). Minimally important difference for the Expanded Prostate Cancer Index Composite (EPIC).

[85] Maly RC, Frank JC, Marshall GN, DiMatteo MR, Reuben DB (1998). Perceived efficacy in patient–physician interactions (PEPPI): validation of an instrument in older persons. J Am Geriatr Soc.

[86] Carver CS (1997). You want to measure coping but your protocol’s too long: Consider the Brief COPE. Int J Behav Med.

[87] Skolarus TA, Wittmann D, Northouse L, An LC, Olson KG, Rew K (2014). Recommendations for prostate cancer survivorship care: an update to the 2009 Michigan Cancer Consortium Guidelines for the Primary Care Management of Prostate Cancer Post-Treatment Sequelae. J Men's Health.

[88] Greenfield S, Cretin S, Worthman LG, Dorey FJ, Soloman NE, Goldberg GA (1981). Comparison of a criteria map to a criteria list in quality-of-care assessment for patients with chest pain: the relation of each to outcome. Med Care.

[89] Greenfield S, Worthman LG, Cretin S (1980). Quality assessment by the criteria mapping method. Top Health Rec Manage.

[90] Piette JD, Rosland AM, Marinec NS, Striplin D, Bernstein SJ, Silveira MJ (2013). Engagement with automated patient monitoring and self-management support calls: experience with 1000 chronically ill patients. Med Care.

[91] Lachin JM (2000). Statistical considerations in the intent-to-treat principle. Control Clin Trials.

[92] Little R, Rubin DB (2000). Causal effects in clinical and epidemiological studies via potential outcomes: concepts and analytical approaches. Annu Rev Public Health.

[93] Curran GM, Bauer M, Mittman B, Pyne JM, Stetler C (2012). Effectiveness-implementation hybrid designs: combining elements of clinical effectiveness and implementation research to enhance public health impact. Med Care.

[94] Damschroder LJ, Aron DC, Keith RE, Kirsh SR, Alexander JA, Lowery JC (2009). Fostering implementation of health services research findings into practice: a consolidated framework for advancing implementation science. Implement Sci.

[95] Janz NK, Mujahid M, Chung LK, Lantz PM, Hawley ST, Morrow M (2007). Symptom experience and quality of life of women following breast cancer treatment. J Women's Health.

