# Supplementary material for: Optimizing veteran-centered prostate cancer survivorship care: study protocol for a randomized controlled trial
Source: Trials. 2017 Apr 18;18:181. doi: 10.1186/s13063-017-1925-4 (PMC5395886; doi:10.1186/s13063-017-1925-4)
Supplement: Supplementary file 1 — SPIRIT Checklist. (DOCX 23 kb) [file 13063_2017_1925_MOESM1_ESM.docx]

**Optimizing Veteran-Centered Prostate Cancer Survivorship Care**

**IVR Call Schedule**

| - **If patient does not answer the call or gets to 1^st^ completion point but doesn’t complete call, the system will continue calling according to this schedule until call is complete or end of call window.** - **Newsletters will be sent following completion of IVR call or after end of call window for all patients making it to 1^st^ completion point.** | | | | |
| --- | --- | --- | --- | --- |
| **Day 1** | **Day 2** | **Day 3** | **Day 4** | **Day 5** |
| IVR calls 2x during preferred times, leaves voicemail after first call | IVR calls 2x during preferred times, leaves voicemail after first call | Study staff make up to 5 or 6 attempts to contact patient (including leaving messages) | IVR calls 4x throughout day, leaves voicemail after first call | Study staff make up to 5 or 6 attempts to contact patient (including leaving messages) |
| AM: 9:30 & 11:30  AFT: 1:30 & 3:30*  EVE: 5:30 & 7:30 | AM: 9:30 & 11:30  AFT: 1:30 & 3:30*  EVE: 5:30 & 7:30 | RA confirms phone # and preferred call time and asks about completing call | 11:30  1:30  5:30  7:30 | RA reminds patient he has until midnight to complete call in order to receive newsletter |
| *AFT will be default call times for those who decline to choose | *AFT will be default call times for those who decline to choose | Options:   - Patient agrees to complete call, hangs up and RA initiates IVR call right away or at a more convenient time later in day - Patient agrees to complete call later and is given phone # **(855-973-0530)** to call when they have time - Patient declines to complete call or withdraws from study   **(For those agreeing to complete call, RA will inform them IVR system will call 4x the next day if call not completed, study staff will contact them again on Day 5, and they have until midnight on Day 5 to complete call.)** |  | Options:   - Patient agrees to complete call, hangs up and RA initiates IVR call right away or at a more convenient time later in day - Patient agrees to complete call later and is given phone # **(855-973-0530)** to call when they have time - Patient declines to complete call or withdraws from study |
