# Supplementary material for: Optimizing veteran-centered prostate cancer survivorship care: study protocol for a randomized controlled trial
Source: Trials. 2017 Apr 18;18:181. doi: 10.1186/s13063-017-1925-4 (PMC5395886; doi:10.1186/s13063-017-1925-4)
Supplement: Supplementary file 2 — IVR call schedule. (DOCX 172 kb) [file 13063_2017_1925_MOESM2_ESM.docx]

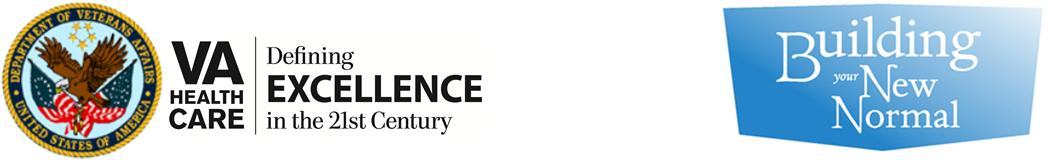


**Building Your New Normal**

**Principal Investigator/Study Chair: Sarah Hawley, PhD**

**EPIC**

This questionnaire asks about four different areas of your prostate health: urinary, bowel, sexual, and overall health. Please think about your experiences over the past **four weeks** when answering each of the following questions. The information contained within this survey will remain strictly confidential. Please do not write your name on this survey. If there any questions you are not comfortable answering, feel free to skip those questions.

**Today's Date**: ____/____/______

**Study ID Number:** _________________

1. Over the **past 4 weeks**, how often have you leaked urine?

More than once a day...................... 1

About once a day............................. 2

More than once a week.................... 3 (Circle one number)

About once a week........................... 4

Rarely or never................................ 5

1. Which of the following best describes your urinary control **during the last 4 weeks**?

No urinary control whatsoever...................... 1

Frequent dribbling......................................... 2 (Circle one number)

Occasional dribbling....................................... 3

Total control................................................... 4

1. How many pads or adult diapers per day did you usually use to control leakage **during the last 4 weeks**?

None ......................................................................... 0

1 pad per day..........................................................… 1

2 pads per day............................................................ 2 (Circle one number)

3 or more pads per day............................................... 3

1. How big a problem if any, has each of the following been for you **during the last 4 weeks**? (Circle one number on each line)

|  | No Problem | Very Small Problem | Small Problem | Moderate Problem | Big Problem |
| --- | --- | --- | --- | --- | --- |
| a. Dripping or leaking urine | 0 | 1 | 2 | 3 | 4 |
| b. Pain or burning on urination | 0 | 1 | 2 | 3 | 4 |
| c. Bleeding with urination | 0 | 1 | 2 | 3 | 4 |
| d. Weak urine stream or incomplete emptying | 0 | 1 | 2 | 3 | 4 |
| e. Need to urinate frequently during the day | 0 | 1 | 2 | 3 | 4 |

1. Overall, how big a problem has your urinary function been for you **during the last 4 weeks**?

No problem...................................... 1

Very small problem.......................... 2

Small problem.................................. 3 (Circle one number)

Moderate problem........................... 4

Big problem...................................... 5

1. How big a problem, if any, has each of the following been for you? (Circle one number on each line)

|  | No Problem | Very Small Problem | Small Problem | Moderate Problem | Big Problem |
| --- | --- | --- | --- | --- | --- |
| a. Urgency to have a bowel movement | 0 | 1 | 2 | 3 | 4 |
| b. Increased frequency of bowel movements | 0 | 1 | 2 | 3 | 4 |
| c. Losing control of your stools | 0 | 1 | 2 | 3 | 4 |
| d. Bloody stools | 0 | 1 | 2 | 3 | 4 |
| e. Abdominal/Pelvic/Rectal pain | 0 | 1 | 2 | 3 | 4 |

1. Overall, how big a problem have your bowel habits been for you **during the last 4 weeks**?

No problem...................................... 1

Very small problem.......................... 2

Small problem.................................. 3 (Circle one number)

Moderate problem........................... 4

Big problem...................................... 5

1. How would you rate each of the following **during the last 4 weeks**? (Circle one number on each line)

|  | Very Poor  to None | Poor | Fair | Good | Very Good |
| --- | --- | --- | --- | --- | --- |
| a. Your ability to have an erection? | 1 | 2 | 3 | 4 | 5 |
| b. Your ability to reach orgasm (climax)? | 1 | 2 | 3 | 4 | 5 |

1. How would you describe the usual QUALITY of your erections **during the last 4 weeks**?

None at all……………………………………………………………………….... 1

Not firm enough for any sexual activity……………………………….2

Firm enough for masturbation and foreplay only…………………3 (Circle one number)

Firm enough for intercourse……………………………………………..…4

1. How would you describe the FREQUENCY of your erections **during the last 4 weeks**?

I NEVER had an erection when I wanted one……………………………...1

I had an erection LESS THAN HALF the time I wanted one…………..2

I had an erection ABOUT HALF the time I wanted one………………..3 (Circle one number)

I had an erection MORE THAN HALF the time I wanted one……..…4

I had an erection WHENEVER I wanted one…………………………........5

1. Overall, how would you rate your ability to function sexually **during the last 4 weeks**?

Very poor.............................................................. 1

Poor...................................................................... 2

Fair....................................................................... 3 (Circle one number)

Good.................................................................... 4

Very good............................................................. 5

1. Overall, how big a problem has your sexual function or lack of sexual function been for you **during the last 4 weeks**?

No problem...................................... 1

Very small problem.......................... 2

Small problem.................................. 3 (Circle one number)

Moderate problem........................... 4

Big problem...................................... 5

1. Which of the following best describes your situation? (Circle one number)

I currently do not have a sexual partner........................ 0 🡪 **GO TO QUESTION 15**

I currently have a sexual partner………........................... 1 🡪 **CONTINUE TO QUESTION 14**

1. Overall, how big a problem has your sexual function been for your partner **during the last 4 weeks**?

Don’t know………………………………….…1

No problem...................................... 2

Very small problem.......................... 3

Small problem.................................. 4 (Circle one number)

Moderate problem........................... 5

Big problem...................................... 6

1. Overall, how big a problem has your sexual function or lack of sexual function been for your ability to date or start new relationships **during the last 4 weeks**?

No problem...................................... 1

Very small problem.......................... 2

Small problem.................................. 3 (Circle one number)

Moderate problem........................... 4

Big problem...................................... 5

1. How big a problem during the last 4 weeks, if any, has each of the following been for you? (Circle one number on each line)

|  | No Problem | Very Small Problem | Small Problem | Moderate Problem | Big Problem |
| --- | --- | --- | --- | --- | --- |
| a. Hot flashes | 0 | 1 | 2 | 3 | 4 |
| b. Breast tenderness/enlargement | 0 | 1 | 2 | 3 | 4 |
| c. Feeling depressed | 0 | 1 | 2 | 3 | 4 |
| d. Lack of energy | 0 | 1 | 2 | 3 | 4 |
| e. Change in body weight | 0 | 1 | 2 | 3 | 4 |
